# Distinct Microbial Communities in Dilated Cardiomyopathy Explanted Hearts Are Associated With Different Myocardial Rejection Outcomes

**DOI:** 10.3389/fcimb.2021.732276

**Published:** 2021-11-29

**Authors:** Jaqueline de Jesus Pereira, Renata Nishiyama Ikegami, Joyce Tiyeko Kawakami, Shérrira Menezes Garavelo, Marcia Martins Reis, Suely Aparecida Pinheiro Palomino, Sandrigo Mangini, Camila Rodrigues Moreno, Samar Freschi de Barros, Aline Rodrigues Souza, Maria de Lourdes Higuchi

**Affiliations:** ^1^ Instituto do Coração (InCor), Hospital das Clínicas HCFMUSP, Faculdade de Medicina, Universidade de São Paulo, São Paulo, Brazil; ^2^ Laboratório de Patologia Cardíaca, Departamento de Patologia, Instituto do Coração (InCor), Universidade de São Paulo, São Paulo, Brazil

**Keywords:** cardiomyopathy, infectious agents, transplantation, myocardial, rejection

## Abstract

**Background:**

Idiopathic dilated cardiomyopathy (IDCM) myocardial inflammation may be associated with external triggering factors such as infectious agents. Here, we searched if moderate/severe heart transplantation rejection is related to the presence of myocardial inflammation in IDCM explanted hearts, associated with microbial communities.

**Method:**

Receptor myocardial samples from 18 explanted hearts were separated into groups according to post-transplant outcome: persistent moderate rejection (PMR; n = 6), moderate rejection (MR; n = 7) that regressed after pulse therapy, and no rejection (NR; n = 5)/light intensity rejection. Inflammation was quantified through immunohistochemistry (IHC), and infectious agents were evaluated by IHC, molecular biology, *in situ* hybridization technique, and transmission electron microscopy (TEM).

**Results:**

NR presented lower numbers of macrophages, as well as B cells (p = 0.0001), and higher HLA class II expression (p ≤ 0.0001). PMR and MR showed higher levels of *Mycoplasma pneumoniae* (p = 0.003) and hepatitis B core (p = 0.0009) antigens. NR presented higher levels of parvovirus B19 (PVB19) and human herpes virus 6 (HHV6) and a positive correlation between *Borrelia burgdorferi* (Bb) and enterovirus genes. Molecular biology demonstrated the presence of *M. pneumoniae*, Bb, HHV6, and PVB19 genes in all studied groups. TEM revealed structures compatible with the cited microorganisms.

**Conclusions:**

This initial study investigating on infectious agents and inflammation in the IDCM explanted hearts showed that the association between *M. pneumoniae* and hepatitis B core was associated with a worse outcome after HT, represented by MR and PMR, suggesting that different IDCM microbial communities may be contributing to post-transplant myocardial rejection.

## Introduction

Idiopathic dilated cardiomyopathy (IDCM), a major cause of heart failure (HF) and heart transplantation (HT), has a still non-clarified etiology, with many theories proposed: viral, autoimmune, genetic, or idiopathic origin (Caforio et al., 2008; Kawai and Matsumori, 2013). Microbial communities have been described in humans and are considered decisive to maintain a healthy situation or to determine diseases (Cho and Blaser, 2012); changes in the composition of microbial community were associated with the worsening of several diseases including HF (Tang et al., 2017).

Patients with HF showed a significantly decreased diversity of the intestinal microbiome as well as a downregulation of key intestinal bacterial groups, pointing to an altered intestinal microbiome as a potential player in the pathogenesis and progression of HF (Luedde et al, 2017). Proteomic and metabolomic analyses of the blood show a distinct immune response and an unexpected link with pathology-specific microbiota in DCM (Seitaro, 2019).

Enterovirus is the most common pathogen observed in DCM, in addition to the human erythrovirus, such as parvovirus B19 (PVB19) (Stewart et al., 2011; Bachelier et al., 2017). Human herpes virus 6 (HHV6) infection is often detected in transplanted and immunosuppressed patients (Ljungman and Singh, 2006), although infections caused by transplant recipients are attributed to asymptomatic reactivation of the virus (Lautenschlager and Razonable, 2012). In addition, bacteria such as *Borrelia burgdorferi* (Bb) (Palecek et al., 2010) and *Mycoplasma pneumoniae* (Koskiniemi, 1993; Nagashima et al., 2010) were found in myocarditis.

In the present work, we characterized and quantified most of the reported antigens/DNA cardiac infectious agents in the DCM explanted heart, focusing on detection of a microbial community related with worse myocardial inflammation, which also could be related with development of persistent myocardial rejection after HT (Felker et al., 2000).

## Methods

### Study Design

Myocardial samples from 18 IDCM explanted hearts were retrospectively studied. Based on medical reports, the patients were grouped according to the degree of rejection (up to 60 days after HT) considering the first episode of no evidence and mild/moderate/severe rejection (meaning 1R, 2R, or 3R), according to the International Society for Heart and Lung Transplantation (ISHLT) revision nomenclature (Stewart et al., 2005), being:

1. Persistent moderate rejection (PMR, n = 6)—myocardial fragments from patients who had PMR
2. Moderate rejection (MR; solved after pulse therapy, n = 7) myocardial fragments from patients who had MR
3. No rejection (NR; or mild rejection, n = 5) myocardial fragments from patients who did not have MR

The fragments were obtained from paraffin blocks of the Laboratory of Pathological Anatomy of the Heart Institute of São Paulo State (InCor-HCFMUSP) and processed for immunohistochemical reactions for detection of antigens, inflammatory cells, and HLA class II expression; *in situ* hybridization (ISH) and qRT-PCR for gene quantification; and transmission electron microscopy (TEM) to identify infectious agents.

This study was approved by the Clinical Hospital of the Medical School of the University of São Paulo Ethics Committee for Analysis of Research Projects under number 3703/11/121.

### Immunohistochemistry

The histological sections used to study the immunohistochemical reaction for Bb (Lot. GR292767-1), *M. pneumoniae* (Lot. P12080102), HHV6 (Lot. FF996), hepatitis C virus (HCV) (Lot. GR202313-2), hepatitis B core (Lot. 10108647) and surface, PVB19 (Lot. 12X11017), enterovirus (Lot. 20028020), CD3 (Lot. 20025164), CD20 (Lot. 20007102), CD45 (Lot. 00048408), CD68 (Lot. M0814), and HLA class II expression (Lot. 20022894) were processed according to the protocol of Tiveron et al. (2017).

### Molecular Biology

#### DNA Extraction and PCR Standardization

DNA extraction from the samples began with tissue dewaxing in xylol baths, followed by dehydration and rehydration in ethanol. After the pellet was obtained, the protocol described by Bettstetter et al. (2003) was followed, with adaptations. The DNA was extracted and purified using PureLink^®^ Viral RNA/DNA Mini Kit (Invitrogen), according to the manufacturer’s specifications. The sample’s concentrations were determined by spectrophotometer reading at wavelength 260/280 nm using NanoDrop apparatus (Thermo Scientific), and the values were expressed in ng/μl. The primers were optimized before being tested on the PCR platform by performing a temperature gradient between 45°C and 68°C. Tissue or culture samples were used as positive controls for the studied agents, and as negative control, ultrapure water was used in place of the samples.

### Cloning *Borrelia* Gene

Cloning of gene Bb was performed according to the protocol of Guilherme et al. (2009) to obtain the positive control to perform the standard curve for real-time PCR quantification.

### Absolute Quantification DNA Copies of Infectious Agents by Real-Time PCR

RT-PCR were performed on 96-well plates using Power SYBR Green Master Mix reagent (Applied Biosystems, USA) as described by the manufacturer and Step One Plus Real-Time PCR Systems equipment (Applied Biosystems). To determine the detection limit of qRT-PCR, standard curves with serial dilutions of extracted DNA were used from samples known to be positive for the genes of interest. All amplifications were terminated with the melting dissociation curve to verify the specificity and amplification and to confirm the absence of primer dimer formation or any other non-specific product.

Absolute quantification was performed for genomic amplification analysis, and all samples were tested in duplicate. The following DNAs sequences were studied: Bb F (GGC AAC CCT AAG GTG AAG GC) and R (GGT GAG CCA GGC CAT CAC TA); HHV6 F (CAA TGC TTT TCT AGC CGC CTC TTC) and R (ACA TCT ATA ATT TTA GAC GAT CCC); *M. pneumoniae* F (ATT ACC ATC CTT GTT GTA AG) and R (GAA GCT TAT GGT ACA GGT TGG); and PVB19 F (TTT CAA AGT CAT GGA CAG TTA TCT GA) and R (TTG TGT AAG TCT TCA CTA GAT AAT ACT GCA TT).

### *In Situ* Hybridization

ISH technique was used to detect *M. pneumoniae* DNA using the biotinylated probe CGTAAGCTATCAGCTACATGGAGG (50 ng/µl). As a positive control, a known positive histological tissue was used for *M. pneumoniae*.

### Transmission Electron Microscopy

Inclusion was performed following the procedure described by Duarte et al. (1992), with adaptations. After selection of the best blocks, 70-nm-thick slices were cut with ultra-microtome and placed in 200 mesh parlodion copper-coated grids (reagent and grids from EMS Electron Microscopy Sciences, Hatfield, PA, USA). Grids were contrasted with lead citrate for analysis in TEM (JEOL JEM-1010; JEOL Ltd).

### Methods of Analysis

After immunohistochemistry (IHC) and ISH processing, slides were scanned using a ScanScope CS System apparatus (Aperio Technologies, Inc., CA, USA) with 40× Olympus UPlanSApo lens. This device was coupled to a scanner that generated image files in.svs format. The scanned images were analyzed using Aperio ImageScope View Software program (Aperio Technologies, Inc., CA, USA). The samples were analyzed using this tool, for viral, bacterial, and HLA class II antigens. For inflammatory cell analysis, the number of positive cells/400× (an average of 20 fields) was quantified to define the average number of immune-stained cells. Results were expressed as cells per mm^2^ (cells/mm^2^), and this number was divided by the value of 0.159, which is the diameter of the microscopic field at ×400 magnification.

### Statistical Analysis

The software GraphPad Prism 6 was used for statistical calculations. Data for each variable were initially compared with the normal curve by the Kolmogorov–Smirnov distance test, being classified as parametric and non-parametric. Parametric data were represented by means and SD, and the groups were compared by ANOVA. Non-parametric data were represented by median and interquartile ranges. The groups were compared by the Kruskal–Wallis test. Spearman’s method was used in the correlation analysis.

## Results

### Inflammatory Profile

**Figure 1:**
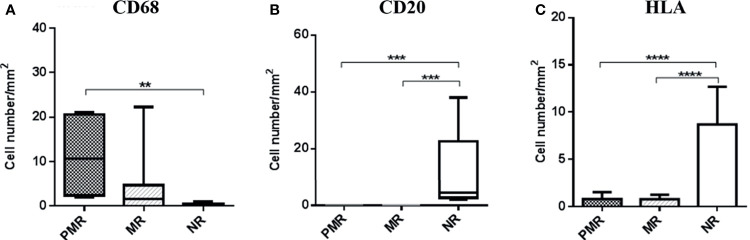
Quantification and comparison of inflammation parameters between groups, demonstrating a hyperactivation of macrophages and infers a better modulation of the immune response in the NR group, by the increase in memory cells. HLA class II expression is also shown to be elevated in this group, validating the result because this molecule also is expressed in B lymphocytes. ** ≤ 0,01; *** ≤ 0,001; **** ≤ 0,0001.

IHC detected higher numbers of CD68+ cells in the PMR group than the two groups (p = 0.005). CD20+ B-cell positive (p = 0.0001) and HLA expression were higher in the NR group (p ≤ 0.0001) (**Figure 1**).

### Infectious Agents by Immunohistochemistry

**Figure 2:**
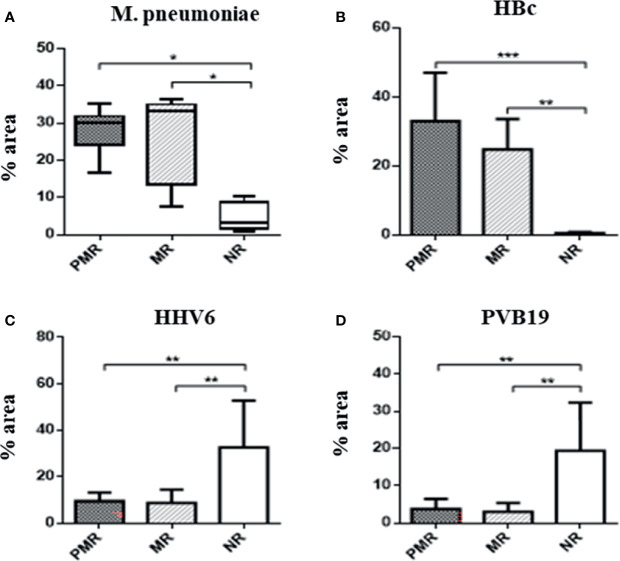
Percentage area of infectious agents in myocardial biopsies by immunohistochemistry. The presence of M. pneumoniae is significant in the groups that show more rejection. This bacteria has ability to modulate the inflammation cells, such as macrophages. * ≤ 0,05; ** ≤ 0,01; *** ≤ 0,001.

There was a predominance of *M. pneumoniae* (p = 0.003) and HBc (p = 0.0009) antigens in the PMR and MR groups, compared with the NR group. In the NR group, there were increased levels of HHV6 (p = 0.01) and PVB19 antigens (p = 0.001) (**Figure 2**). Data obtained from all infectious antigens analyzed were expressed in the **Table 1**.

**Table 1:** Data obtained from quantification of the microorganisms by immunohistochemistry technique.

	PMR	MR	NR	P
**Bb**	10.1	12.2	14	0.8
	(7 – 15.3)	(5.6 – 15.2)	(6.6 – 23.4)	
**HBc**	31.6	21.7	0.8	0.0009
	(20.8 – 44.2)	(18.8 - 30)	(0.3 – 0.8)	
**HBs**	1.15	1.7	1.5	0.9
	(0.4 – 5.7)	(0.8 - 2)	(0.7 – 4.6)	
**HCV**	14	13	32	0.08
	(11.8 – 20.7)	(10.8 – 20.8)	(17.2 – 47.5)	
**HHV6**	10.2	8.8	30	0.01
	(6.1 – 12.5)	(4.3 – 10.8)	(16.2 – 50.2)	
**Enterovirus**	32.6	25.03	33.2	0.3
	(24.2 – 37.2)	(18.7 – 31.4)	(18.9 – 36.3)	
***M. pneumoniae***	29.9	33.1	3.3	0.003
	(24.1 – 31.8)	(13.6 - 35)	(1.6 – 8.6)	
**PB19**	3.2	2.3	18.2	0.001
	(1.5 – 5.8)	(0.8 – 5.5)	(9.1 – 30.1)	

PMR, persistent moderate rejection; MR, moderate rejection; NR, no rejection; Bb, Borrelia burgdorferi; HCV, hepatitis C virus; HHV6, human herpes virus 6; PVB19, parvovirus B19.

### Infectious Agents by Real-Time PCR

**Figure 3:**
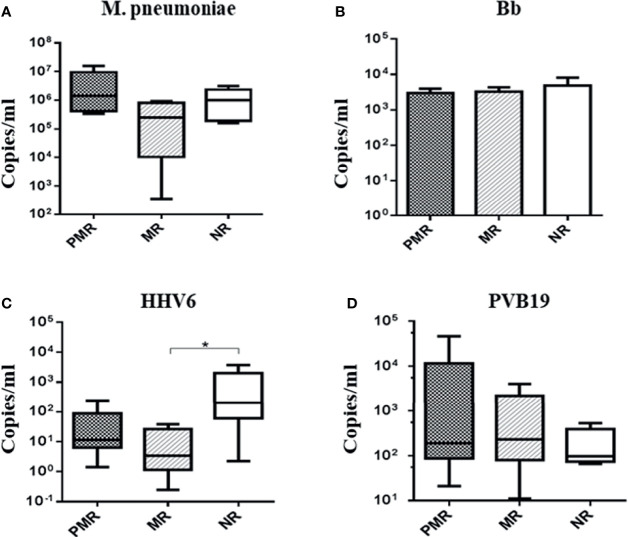
Absolute quantification of infectious agents in myocardial biopsies. * Comparison MR × NR group: p < 0.05: HHV6.

The presence of genomes from *M. pneumoniae* and Bb bacteria, and from HHV6 and PVB19 viruses, was evident in all patients (**Figure 3**). There was a higher amount of HHV6 gene in the NR versus MR group (p = 0.05).

### *Mycoplasma pneumoniae* by *In Situ* Hybridization

**Figure 4:**
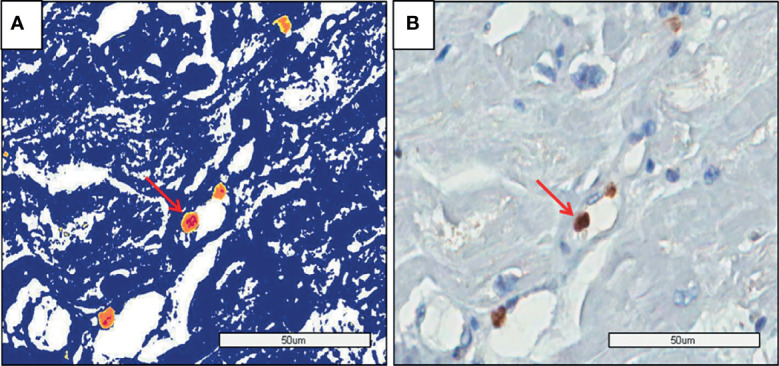
PMR myocardium: **(A)** The image shows the genome identification (yellow and red dots) by Aperio analysis. **(B)** The image shows the genome identification without the analysis method by software (Aperio).

There was no difference in the amount of DNA among the groups (**Table 2**). **Figure 4** shows genome identification in myocardial tissue.

**Table 2:** Presence of the *Mycoplasma pneumoniae* genome in the groups.

	PMR	MR	NR	p
ISH *M. pneumoniae*	0.75 (0.6–2.3)	1.35 (1.3–2.1)	3.3 (0.55–4.1)	0.44

PMR, persistent moderate rejection; MR, moderate rejection; NR, no rejection; ISH, in situ hybridization.

### Correlation Between Microorganisms

The amount of *M. pneumoniae* DNA had an almost significant negative correlation with PVB19, Bb, and HCV antigens in the PMR group. In the NR group, the Bb DNA correlated with enterovirus antigen (**Table 3**).

**Table 3:** Correlation of antigen expression and molecular biology.

	PMR		NR	
	r	p	r	p
***Mycoplasma pneumoniae* × PVB19**	−0.7	0.06		
***M. pneumoniae* × HBs**	−0.7	0.06	−0.9	0.08
***M. pneumoniae* × Bb**	−0.9	0.06		
***M. pneumoniae* × HCV**	−0.9	0.08		
**Bb × Enterovirus**			1	0.01

PMR, persistent moderate rejection; NR, no rejection; PVB19, parvovirus B19; HCV, hepatitis C virus; Bb, Borrelia burgdorferi.

### Transmission Electron Microscopy

**Figure 5:**
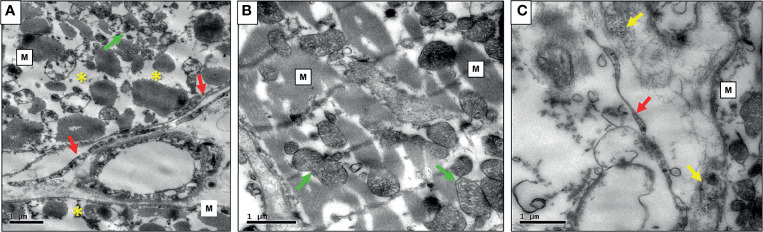
**(A)** Photomicrography of PMR case exibiting myocardial fibers (M) with severe myocytolysis (*) and mitochondria with cristolysis (green arrow), large and long borrelias at the extracelular matrix (red arrows); **(B)** Mild rejection case with myocardial fibers well-preserved (M), mithocondria without myocytolysis (green arrows), and mild intracellular edema; **(C)** NR case exibiting thinner and smaller Borrelia (red arrow) in the interstitium, Mycoplasma (M) and Enterovirus (yellow arrows).

Also, some cases in which high myocardial tissue antigenic expression of infectious agents was found by IHC were studied by electron microscopy, which revealed morphologies compatible with microorganisms in symbiosis, as demonstrated in **Figures 5A–C**.

## Discussion

Different microbiomes may explain a chronic inflammatory state, a more pathogenic microbiome related with more inflammation, and severity of the disease (NIH, 2021). The loss of cardiac mass that may lead to enlargement of the ventricular chamber and the thinning of ventricular walls is highly correlated with HF and mortality (Harvey and Leinwand, 2011).

In the present study, a morphomolecular characterization and quantification of *M. pneumoniae*, Bb, HHV6, HBs, HBc, HCV, and PVB19 in the explanted hearts of patients with DCM detected different microbiomes. Previously, we have already detected a high incidence of Bb, HHV6, and HBc antigens in patients with DCM, suggesting a causal relationship of these microorganisms in the pathogenesis of the disease (Mangini et al., 2015). HF in Chagas disease was also associated with microorganisms in symbiosis (Higuchi et al., 2009). Other groups also described contribution of microbial communities in the pathogenicity and worse prognosis of the diseases (Dessì et al., 2005; Hansen et al., 2007; Schwabe and Jobin, 2013).

In explanted hearts of the group had an outcome of PMR, there was higher expression of *M. pneumoniae* antigens and macrophages, in accordance with the findings of Nakayama et al. that excessive immune activation in the myocardium mediated by T lymphocytes and macrophages was associated with a worse prognosis in patients with DCM (Nakayama et al., 2017). A negative correlation between the amount of *M. pneumoniae* with Bb, HCV, PVB19, and HBs suggest that a microbial community having larger numbers of infectious agents in symbiosis favors increased inflammation and increased capacity of these agents to remain in the patients and to develop persistent myocardial inflammation in the donor heart after CT.

Classically, mycoplasmas act as extracellular parasites, and the pathogenicity is dependent on the attachment sites and the onset of injury to the host cell membrane (Collier and Clyde, 1971). Their presence may stimulate hyperactive macrophages and activate natural killer cells, in addition to stimulating the proliferation of T and B cells and expression of class I and II molecules of the major histocompatibility complex inducing fibroblast apoptosis (Razin et al., 1998; Yang et al., 2002). Although *M. pneumoniae* is classically considered an extracellular pathogen, in certain circumstances such as low immune system resistance or increased pathogenicity of the infectious agents, this bacterium can live and replicate intracellularly in human cells (Baseman et al., 1995; Yavlovich et al., 2004). Residence in an intracellular reservoir may explain its ability to establish chronic infection, as what occurs in asthmatic patients, possibly shielded from the host’s immune response and antibiotic action (Martin et al., 2001; Hardy et al., 2002).

Our correlation findings indicate an intimate relationship between *M. pneumoniae* and Bb in the PMR group, possibly leading to their increased virulence, represented by higher numbers of active macrophages. Mycoplasmas have the ability of stimulating macrophage proliferation (Harvey and Leinwand, 2011; Mangini et al., 2015). Favoring this hypothesis, the PMR group was the only group to present correlations between mycoplasma and different viruses, probably increasing the pathogenicity of this bacterium to live and replicate intracellularly in human cells (Hansen et al., 2007; Higuchi et al., 2009) and controlling virus proliferation, thereby explaining negative correlations. Although p-values sometimes were not statistically significant, we consider these results important for discussion.

Studies have shown that DCM may result from viral myocarditis due to the persistence of viruses in cardiomyocytes (Lotze et al., 2004; Moimas et al., 2012). HHV6 can infect cell types like monocytes, macrophages, fibroblasts, and endothelial cells (Stewart et al., 2011). After the primary infection, HHV6 persists in the latency state in the host, where the viral genome is harbored as circular DNA in several cells, and can be reactivated later, especially during periods of immunosuppression (Stewart et al., 2011; Seitaro, 2019). In addition to enteroviruses, PVB19 has been associated with the pathogenesis of myocarditis; however, these findings are controversial because this virus can also be found in the heart of healthy individuals (Verdonschot et al., 2016).

HCV development-associated cardiomyopathy can occur in susceptible patients in whom the viral and immunological mechanisms can produce myocardial and cardiovascular damage, such as plaque enlargement in the carotid arteries (Fukui et al., 2003; Sanchez and Bergasa, 2008). In addition, a recent study showed that HCV-positive patients had higher mortality rates for cardiovascular diseases than HCV-negative patients (Cacoub et al., 2016), evidencing the importance of this virus in heart disease. However, none of these studies considered the possibility of a microbiome containing mycoplasma and viruses.

The MR group did not have a correlation between microorganisms, suggesting that when there is no symbiotic coinfection, the infectious agents are more susceptible to pulse therapy.

Coinfection with *M. pneumoniae* may exacerbate Lyme disease by modulating the immune system, and this may be one of the causes of resistance to therapy by the synergistic-pathological mechanism (Berghoff, 2012). The prevalence of Bb in cardiac involvement has been increasingly evident, being detected in endomyocardial samples of patients with suspected inflammatory heart disease (Karatolios et al., 2015). Furthermore, similar findings have suggested that Central Europe represents an endemic area with a relatively high frequency of DCM related to Bb (Kubánek et al., 2012).

In the NR group, high numbers of HHV6 antigens and genes, high numbers of PVB19 antigens, and a low percentage of *M. pneumoniae* antigens associated with an increase in the mean numbers of B cells and higher HLA class II expression suggest that this group has the most effective immune system, resulting in better control of microorganisms.

This work shows that despite the high prevalence of HHV6 and PVB19 in the NR group, the coinfection without the participation of microorganisms such as *M. pneumoniae* stimulates an immune response more effectively, controlling the virus’s infection and consequently helping to prevent the development of MR (Alizon and van Baalen, 2008; Mideo, 2009).

Microbiome presenting an interaction between different species of microorganisms, for example, in our present work of mycoplasma, borrelia, and viruses, leads to the development of more resistant and productive symbiotic forms, than the isolated forms as described in the literature (NIH, 2021). The presence of microorganisms as host symbionts is an important aim of the search for an explanation of the theory of infection; thus, it is an increasingly studied subject (Hussa and Goodrich-Blair, 2013). Also, we intend to continue this research in order to clarify if microbiome represented by symbiosis predominantly between bacteria *M. pneumoniae* and *B. burgdorferi* with viruses induces release of small extracellular vesicles (sEVs) associated with abnormal cellular immune response, as described in the literature (Christodoulides et al., 2018), and if these are present in the rejection episodes in morphomolecular studies of biopsies after CT (Govender et al, 2020). These studies may support the concept that symbiosis with viruses may provide benefit to the main symbiont by exploiting sEVs as a vehicle for intercellular communications and modifying host immune response activation.

## Conclusion

Microbiome formed by bacteria and virus close interaction may be the causal and/or perpetuating factor of myocardial injury in patients with idiopathic DCM. Our initial findings, in a small number of cases, suggest that the morphomolecular characterization of microorganisms and inflammation in the myocardium of explanted hearts in CT patients may indicate different prognoses regarding intensities of myocardial rejection episodes. Further studies with increased number of patients and that analyze the endomyocardial biopsies in the follow-up of CT are necessary.

### Study Limitations

We are aware of the limitations of the study, mainly in relation to the size of the sample. However, due to the importance of the findings, and to support the idea that the presence of more virulent microbiome associated with inflammation could be influencing the pathogenesis of DCM, we intend to evaluate endomyocardial follow-up biopsy to verify the findings that infections present in the explanted hearts as we have demonstrated resurge in the donor’s heart and are associated with episodes of rejection.

## Data Availability Statement

The raw data supporting the conclusions of this article will be made available by the authors, without undue reservation.

## Ethics Statement

The studies involving human participants were reviewed and approved by the Clinical Hospital of the Medical School of the University of São Paulo Ethics Committee for Analysis of Research Projects under number 3703/11/121. The patients/participants provided their written informed consent to participate in this study. Written informed consent was obtained from the individual(s) for the publication of any potentially identifiable images or data included in this article.

## Author Contributions

JP and MH: conceptualization. JP, RI, JK, CM, SP, SB, and AS: performance of laboratory examinations. JP, MH, JK, SP, and MR: interpretation of data. JP and MH: performance of statistical analyses. JP, MH, SG, and RI: writing and original manuscript draft preparation. JP, SM, and MH: contribution of clinical data. JP, MH, SG, and RI: manuscript review and editing. All co-authors participated in the formulation of the manuscript or critically reviewed it, contributed to its intellectual content, approved the version to be published, and agreed to be responsible for all aspects of the work, ensuring that issues related to the accuracy or integrity of any part of the work were properly investigated and resolved.

## Funding

This study was supported by Fundação de Amparo à Pesquisa do Estado de São Paulo (FAPESP) and Coordenação de Aperfeiçoamento de Pessoal de Nível Superior (CAPES) numbers 2015/25786-2 and 1729745.

## Conflict of Interest

The authors declare that the research was conducted in the absence of any commercial or financial relationships that could be construed as a potential conflict of interest.

## Publisher’s Note

All claims expressed in this article are solely those of the authors and do not necessarily represent those of their affiliated organizations, or those of the publisher, the editors and the reviewers. Any product that may be evaluated in this article, or claim that may be made by its manufacturer, is not guaranteed or endorsed by the publisher.
